# Insulin-Like Growth Factor-I Induces Arginase Activity in *Leishmania amazonensis* Amastigote-Infected Macrophages through a Cytokine-Independent Mechanism

**DOI:** 10.1155/2014/475919

**Published:** 2014-09-09

**Authors:** Celia Maria Vieira Vendrame, Marcia Dias Teixeira Carvalho, Andre Gustavo Tempone, Hiro Goto

**Affiliations:** ^1^Laboratório de Soroepidemiologia e Imunobiologia, Instituto de Medicina Tropical de São Paulo, Universidade de São Paulo, Avenida Dr. Enéas de Carvalho Aguiar 470, Predio II, 4° Andar, 05403-000 São Paulo, SP, Brazil; ^2^Laboratório de Imunofisiopatologia, Instituto de Ciências Biomédicas IV, Universidade de São Paulo, 05508-900 São Paulo, SP, Brazil; ^3^Departamento de Parasitologia, Instituto Adolfo Lutz, 01246-903 São Paulo, SP, Brazil; ^4^Departamento de Medicina Preventiva, Faculdade de Medicina, Universidade de São Paulo, 01246-903 São Paulo, SP, Brazil

## Abstract

*Leishmania (Leishmania) amazonensis* exhibits peculiarities in its interactions with hosts. Because amastigotes are the primary form associated with the progression of infection, we studied the effect of insulin-like growth factor (IGF)-I on interactions between *L. (L.) amazonensis* amastigotes and macrophages. Upon stimulation of infected macrophages with IGF-I, we observed decreased nitric oxide production but increased arginase expression and activity, which lead to increased parasitism. However, stimulation of amastigote-infected macrophages with IGF-I did not result in altered cytokine levels compared to unstimulated controls. Because IGF-I is present in tissue fluids and also within macrophages, we examined the possible effect of this factor on phosphatidylserine (PS) exposure on amastigotes, seen previously in tissue-derived amastigotes leading to increased parasitism. Stimulation with IGF-I induced PS exposure on amastigotes but not on promastigotes. Using a PS-liposome instead of amastigotes, we observed that the PS-liposome but not the control phosphatidylcholine-liposome led to increased arginase activity in macrophages, and this process was not blocked by anti-TGF-*β* antibodies. Our results suggest that in *L. (L.) amazonensis* amastigote-infected macrophages, IGF-I induces arginase activity directly in amastigotes and in macrophages through the induction of PS exposure on amastigotes in the latter, which could lead to the alternative activation of macrophages through cytokine-independent mechanisms.

## 1. Introduction

Leishmaniases are diseases with tegumentary or visceral forms that are caused by protozoans of the genus* Leishmania *and are transmitted via insect vectors and affect more than 12 million people in tropical and subtropical areas of the world [1].* Leishmania (Leishmania) amazonensis* is one of the most prevalent species that causes tegumentary leishmaniasis in the New World causing localized cutaneous, mucosal, or diffuse cutaneous leishmaniases [2]. The control or progression of leishmaniasis in the vertebrate host depends on nonspecific and specific factors of the immune response as well as the ability of the parasite to evade the host response [3]. However, the various disease manifestations and outcomes may result from diverse* Leishmania* species-related immune and immunopathogenic responses and* L. (L.) amazonensis* exhibits peculiarities that distinguish it from other species.

The differences in the immunopathogenic mechanisms of patients infected with either* L. (Viannia) braziliensis* or* L. (L.) amazonensis*, the species that are prevalent in Brazil, are evident. While the response to* L. (V.) braziliensis* infection is related to the exacerbation of the Th1-type response [4–6] or to defects in the modulation of IFN-*γ* production or decreased IL-10 receptor expression in severe mucosal cases [6, 7],* L. (L.) amazonensis* infection tends to elicit a less effective or limited T cell-response against the parasite [5, 8], which could explain the development of diffuse cutaneous leishmaniasis in certain cases. These differences are also noticeable in cutaneous leishmaniasis in mice. Compared to* L. major* infection, to which the BALB/c mouse strain is susceptible and the C57BL/6 strain is resistant [9], both mouse strains are resistant to* L. (V.) braziliensis* [10] and both are susceptible to* L. (L.) amazonensis* [11]. The development of lesions in* L. (L.) amazonensis*-infected mice is not only dependent on the Th2 immune response [12] but rather on the presence of Th1 CD4^+^ T cells [13] and IFN-*γ*, which promotes parasite growth in amastigote-infected macrophages in vitro [14]. The diverse outcomes and development of specific immune responses during* L. (L.) amazonensis* infection could be attributed to alterations observed during early innate* Leishmania*-host interactions involving inflammatory cytokines, chemokines, Toll-like receptors, dendritic cell activation, and so forth [15]. Thus in the present study, we focused on the effect of a growth factor in the nonspecific interaction between* L. (L.) amazonensis* and macrophages.

Among nonspecific elements, we have been studying the effect of insulin-like growth factor (IGF)-I on* Leishmania* and in leishmaniases. IGFs are polypeptides with a molecular mass of approximately 7.5 kDa, that exist in two major forms, IGF-I and IGF-II, which share 60% similarity and are found in circulation associated with IGF binding proteins and in tissues. IGFs affect cell metabolism and are important endocrine growth and differentiation factors [16]. Based on these characteristics, we previously studied the effect of IGFs on leishmaniasis during their initial interactions as nonspecific factors, though the Th1 cytokine IFN-*γ* is known to inhibit IGF-I [17], and the Th2 cytokines IL-4 and IL-13 to increase IGF-I expression [18].

We have previously shown that adding physiological concentrations of extrinsic IGF-I into cultures induces proliferation of different species of* Leishmania* promastigotes and axenic amastigotes, which is an effect that is not observed with IGF-II despite its high similarity to IGF-I [19, 20]. In experimental models, extrinsic IGF-I significantly increases lesion sizes and the number of viable parasites [21].

During infection however, the interaction between amastigotes and host cells is critical for the progression of infection and the establishment of disease, which is different to promastigotes. This fact was elegantly proven recently using axenic* Leishmania major* amastigotes [22]. Specifically, comparing* Leishmania (L.) amazonensis* promastigotes and amastigotes showed differences in dendritic cell activation [22], parasitism after IFN-*γ* stimulation [14], susceptibility to histone proteins [23], and intracellular signaling after IGF-I stimulation [24]. Therefore, in the present study, we analyzed the effects of IGF-I on the interaction between* Leishmania (L.) amazonensis* amastigotes and macrophages.

Because our previous data show that IGF-I favors parasite growth and arginase activation using* Leishmania (L.) amazonensis* promastigotes [25], we initially evaluated parasitism and arginase activity on amastigote-infected macrophages and on cell-free-amastigotes upon IGF-I stimulation. Then, we analyzed inflammatory and Th1 and Th2 cytokine production in infected macrophages upon IGF-I stimulation because of their potential role in macrophage activation [26]. Another phenomenon related to the progression of* L. (L.) amazonensis *infection is the phosphatidylserine (PS) exposure that was observed only on tissue-derived amastigotes [27–29]. Because no induction factor was identified before and IGF-I is present in tissue fluids, we evaluated PS exposure upon IGF-I stimulation and the possible role of the PS molecule on macrophage activation.

In the present study, we thus show that IGF-I induces arginase activity directly in* Leishmania (L.) amazonensis* amastigotes and in amastigote-infected macrophages, the latter through the induction of PS exposure on amastigotes that could lead to the alternative activation of macrophages through cytokine-independent mechanisms.

## 2. Material and Methods

### 2.1. Parasites

*Leishmania (Leishmania) amazonensis* (MHOM/BR/73/M2269) was kindly provided by Professor Clara Lucia Barbieri from Universidade Federal de São Paulo and was maintained through regular passage inBALB/c mice. Amastigotes were obtained from footpad lesions of BALB/c mice infected one month earlier. The lesions were aseptically dissected and washed in 0.01 M phosphate-buffered saline pH 7.4 (PBS) and cut and ground in a Petri dish containing PBS. Then, the suspension was disrupted by four passages through 22-gauge needles and centrifuged 3 times at 250 ×g for 10 min; the resulting supernatant was centrifuged at 1,400 ×g for 30 minutes and the parasites were suspended in RPMI 1640 medium (Gibco, Carlsbad, CA, USA). The purified amastigotes were used immediately in the experiments or were expanded to promastigotes and maintained in 199 medium (Gibco) supplemented with 10% heat-inactivated fetal calf serum (Cultilab, Campinas, SP, Brazil) at 26°C. The promastigotes used in these experiments were in the stationary-phase of growth and had undergone no more than four passages in culture.

### 2.2. Mice

BALB/c mice of both sexes, aged 6–8 weeks, were obtained from the Animal Facility of the Faculdade de Medicina da Universidade de São Paulo and used as a source of peritoneal macrophages or for the in vivo maintenance of* L. (L.) amazonensis.* This study was approved by the Ethical Committee for Animal Research (CEEA), at Biomedical Sciences Institute/Universidade de Sao Paulo, protocol number 041/03, adhering to the Ethical Principles in Animal Research adopted by Brazilian College of Animal Experimentation (COBEA).

### 2.3. Macrophage Culture, Infection and Stimulation

Resident peritoneal cells were obtained from the peritoneal cavities of BALB/c mice and suspended in RPMI 1640 medium supplemented with 100 UI/mL penicillin, 100 *μ*g/mL streptomycin, and 2% heat-inactivated BALB/c mouse serum (complete medium). The concentration was adjusted to 4-5 × 10^6^ cells/mL and the cells were plated in different amounts either on round 13 mm^2^glass cover slips placed in the wells of 24-well plates (Corning Life sciences Tewksbury, MA, USA) or directly into the wells and incubated for 2 h to adhere on the plate at 37°C in a humidified atmosphere containing 5% CO_2_. Thereafter, the wells were washed twice with RPMI 1640 medium to remove any nonadherent cells. Then, either adherent cells (macrophages) or* Leishmania* amastigotes or promastigotes were preincubated for 5 minutes with 50 ng/mL recombinant human IGF-I or IGF-II (R & D Systems, Minneapolis, MN, USA) and then washed out. The parasite suspension (2 parasites/1 cell ratio) was dispensed into the wells and allowed to infect for 2 h at 33°C in a humidified atmosphere containing 5% CO_2_ and then the noninternalized parasites were washed out and the culture was maintained at 33°C in a humidified atmosphere containing 5% CO_2_. In some cases, the IGF-I and IGF-II were maintained throughout the experimental period. Controls were maintained without IGF-I. In some experiments, 100 *μ*M N^G^-hydroxy-L-arginine acetate salt (NOHA) (Sigma-Aldrich Co., St. Louis, MO, USA) was added to the culture or used to preincubate the amastigotes for 5 minutes and was washed out [30]. Tests were run in sextuplicate and incubated for 2, 24, 48, or 72 h. The coverslips, supernatants, or cell lysates were recovered for different evaluations. In the experiments for the evaluation of H_2_O_2_ production, phenol red free medium was used.

### 2.4. Parasite Burden in Macrophages

The cover slips were removed from the plates, mounted and stained with Giemsa dye and processed for the evaluation of parasitism under a light microscope (Carl Zeiss, Göttingen, Germany). Six hundred cells were counted for each treatment condition. The data are presented as the number of parasites in 100 cells from the formula [(number of parasites: number of infected cells) × (number of infected cells/total number of cells) × 100]. This analysis was performed by two independent observers who were blinded to the experimental conditions.

### 2.5. Cell and Parasite Lysates: Sample Preparations

Four million cells in 500 *μ*L were plated in 24-well culture plates and infected and stimulated with IGF-I as described previously. Some sets were stimulated with IFN-*γ* (10 ng/mL) or lipopolysaccharide (LPS) from* Escherichia coli* (Sigma Co., Ltd. (St Louis, MO, USA)) (1 *μ*g/mL) or NOHA (100 *μ*M). After 24 h of incubation, the cells were washed twice with ice-cold PBS and then lysed for 10 minutes with ice-cold lysis buffer (10 mM Tris-HCl pH 7.6, 150 mM NaCl, 2% NP-40 substitute (octylphenoxypolyethoxyethanol), 1 mM 4-(2-aminoethyl)-benzenesulfonyl fluoride and 5 *μ*M leupeptin). The lysed cell preparation was centrifuged at 10,000 ×g for 5 minutes at 4°C and the protein concentration in the supernatant was determined using a Lowry protein assay [31] to adjust the concentrations before analysis. These lysates were used in western blot analyses and arginase expression and activity assays. For the analysis of* Leishmania* amastigote arginase activity, the same lysate preparation protocol was used.

### 2.6. SDS-PAGE and Western Blot Analysis

Cell lysates (20 *μ*g protein in 20 *μ*L) were run on sodium dodecyl sulphate-10% polyacrylamide gels. The separated proteins were electrotransferred onto 0.2 mm pore size nitrocellulose membranes using transblot SD-semidry transfer cells (Bio Rad, CA, USA; 30 minutes at 15 mV). The membranes were blocked with 150 mM NaCl, 20 mM Tris, 0.01% Tween 20, pH 7.4 buffer (TBS-T buffer) containing 5% fat-free milk for one hour. The membranes were reacted with a monoclonal anti-arginase-I antibody (1 : 1000 in PBS) (BD Biosciences Pharmigen, San Diego, CA, USA) for 2 h at room temperature, washed three times with TBS-T and incubated with a peroxidase-conjugated polyclonal anti-mouse IgG (1 : 1000) (Sigma-Aldrich, Saint Louis, MO, USA) for one hour at room temperature. Rainbow protein molecular weight markers (Bio-Rad Laboratories, Hercules, CA, USA) were used. Bound antibodies were detected by an ECL chemiluminescence kit (Amersham Biosciences, Piscataway, NJ, USA) following the manufacturer's instruction.

### 2.7. Arginase Activity

Cells and amastigotes were taken from the culture and the obtained lysates were assayed for arginase activity as described previously [32]. Briefly, to activate the arginase, 50 *μ*L of the lysates were treated with the same volume of 5 mM MnCl_2_, 25 mM Tris-HCl pH 7.4 at 56°C for 10 min. Then, to 25 *μ*L of the activated lysate, 25 *μ*L of 0.5 M L-arginine pH 9.7 were added and incubated at 37°C for 60 min. The reaction was stopped with 400 *μ*L of H_2_SO_4_/H_3_PO_4_/H_2_O (1/3/7, v/v/v). The urea concentration was measured at 540 nm in a Multiskan MCC/340 P version 2.20 plate reader spectrophotometer (Labsystems, Vantaa, Finland) after the addition of 25 *μ*L of 9% *α*-isonitrosopropiophenone in 100% methanol and incubation at 100°C for 45 min. One unit of enzyme activity is defined as the amount of enzyme that catalyzes the formation of 1 *μ*mol of urea per minute.

### 2.8. Detection of Nitrite

Nitrite (NO_2_^−^) accumulation in the cell culture supernatants was used as an indicator of nitric oxide production and it was determined by the Griess reaction [33]. The absorbance of the reaction product at 570 nm was measured using an ELISA reader. The nitrite concentration was determined using sodium nitrite as a standard.

### 2.9. Detection of**  **H_2_O_2_

The assay is based on the horseradish peroxidase (HRPO)-mediated oxidation of phenol red by H_2_O_2 _as described by Pick and Keisari [34]. A standard curve was established using a H_2_O_2_ solution of known concentration. Phorbol myristate acetate (PMA) 2 mM (Sigma Co., Ltd., St Louis, MO, USA) was used as positive control. Tests were run in sextuplicate.

### 2.10. Measurement of****Cytokines

The cell culture supernatants were harvested after 24 h for the analysis of IL-1*β*, 48 h for IL-6 and TNF and 72 h for TGF-*β* and IFN-*γ* by ELISA according to the manufacturer's instructions using BD OptEIA kits (BD Biosciences, USA) in 96-well tissue culture plates (Corning Costar Co., USA) and read at 450 nm in a Multiskan MCC/340 P version 2.20 spectrophotometer (Labsystems, Vantaa, Finland). The sensitivity of the cytokine assays was as follows: IFN-*γ*, 8 pg/mL; IL1-*β*, 8 pg/mL; IL-6, 16 pg/mL; TGF-*β*, 20 pg/mL; and TNF, 8 pg/mL. The concentrations were determined by comparison with a curve generated from each cytokine standard. All of the cytokine measurements were performed in triplicate and at the same time to avoid intertest variations.

### 2.11. Measurement of Prostaglandin E_2_ (PGE_2_)

The assay is based on the competitive binding technique in which the PGE_2_ present in the supernatant competes with a fixed amount of alkaline phosphatase-labeled PGE_2_ for sites on mouse monoclonal anti-PGE_2_ antibodies. The analysis was performed according to the manufacturer's instructions using a Prostaglandin E_2_ immunoassay (R&D System, Inc. Minneapolis, MN, USA). The sensitivity of the PGE_2_ assay was 15.9 pg/mL. The concentrations were determined by comparison with a standard curve.

### 2.12. Effect of**  **IGF-I on Phosphatidylserine-Exposure

Annexin V binds to negatively charged phospholipid surfaces with a higher specificity for PS. The detection of annexin V was made by flow cytometry after staining with fluorescein isothiocyanate-conjugated annexin V and propidium iodide (PI) using a commercially available kit (Annexin V FITC Apoptosis Detection KIT II-BD Biosciences). Amastigotes (10^6^ parasite/mL) were treated in the same way as described above with 50 or 100 ng/mL IGF-I or with H_2_O_2_ (4 or 8 mM) [35] as a positive control for 24 h. Then, the cells were washed twice with cold PBS and 100 *μ*L of the solution (10^5^cells) was resuspended in annexin V binding buffer (10 mM HEPES, 140 mM NaCl, 2.5 mM CaCl_2_; pH 7.4) and incubated with 5 *μ*L of annexin V FITC and 5 *μ*L of PI at 4°C for 15 minutes in the dark. After this period, 400 *μ*L of binding buffer was added to each tube and the cells were collected in a BD FACSCalibur (Becton Dickinson, Franklin, NJ, USA) and analyzed by Cell Quest. A total of 10,000 events were harvested from each sample. We evaluated annexin V positive and PI negative populations.

### 2.13. Effect of the Negatively Charged Lipid PS-Liposome on Arginase Activity of BALB/c Macrophages

#### 2.13.1. Liposome Preparation

Liposomes were prepared by the film-hydration method, using hydrogenated phospholipids (LIPOID Gmbh, Germany) [36]. For the preparation of the negatively charged liposomes (PS-liposomes), saturated egg phosphatidylcholine, saturated egg phosphatidylserine, and cholesterol (Sigma-Aldrich) (7 : 2 : 1 molar ratio) or neutral liposomes (PC-liposomes-control group), phosphatidylcholine, and cholesterol (9 : 1) were dissolved in chloroform : methanol (1 : 1 ratio). The solution was further sonicated for 10 minutes. The mixture was evaporated in a rotary evaporator at 55°C at 60 rpm for 40 minutes in a vacuum and protected from light. A preheated (55°C) solution of 2.25% glycerol (9 mL) was added to the lipid film using glass beads. The swelling process of the preformed liposomes was performed in a rotary evaporator at 55°C at 80 rpm for 60 minutes without a vacuum. The surface charge of neutral and negatively charged liposomes was previously confirmed using the zeta potential analysis, in a Zeta Plus Analyzer (BrookHaven Instr. Corp.). The samples were diluted in 1 mL of 3 mM KCl as recommended by the manufacturer. The diluted samples were then analyzed for ten cycles with a voltage of 4 mV. The phospholipid content of the liposomal formulations was determined by the Stewart assay [37].

#### 2.13.2. Arginase Activity with Liposome

Macrophages (2 × 10^6^/500 *μ*L RPMI 1640 medium) were plated in a 24-well plate and incubated for 2 h to allow adherence at 37°C in a humidified atmosphere containing 5% CO_2_. Thereafter, the wells were washed twice with RPMI 1640 to remove any nonadherent cells. Then, we infected some wells with amastigotes (2 parasites/1 cell ratio) or used 30 *μ*L of phosphatidylcholine- (PC-) liposome 30%, 30 *μ*L of PS-liposome 30%, or 30 *μ*L of 2.25% glycerol as stimuli. Unstimulated macrophages were used as a control. We performed the same experiment in parallel using 3 *μ*L of an anti-TGF-*β* antibody (15 *μ*g/mL) in each well (R&D Systems, Inc., USA). After 24 h, the cells were treated to obtain the lysates and the arginase activity reaction was performed as described above.

### 2.14. Statistical Analysis

The statistical analyses were performed using GraphPad Prism 5 (GraphPad Software, Inc., San Diego, CA, USA). The data were analyzed by ANOVA with the Student Newman-Keuls contrast post-test and were considered significant when *P* < 0.05. The cytokine absorbances were analyzed after transformation and linear regression. The results are expressed as medians and percentiles (25–75). The Kruskal-Wallis test with the Student Newman-Keuls contrast post-test was used for statistical comparisons among groups using SigmaStat for Windows Version 3.10 (Systal Software, Inc., San Jose, CA, USA). A 0.05 confidence level was considered significant.

## 3. Results

**Figure 1:**
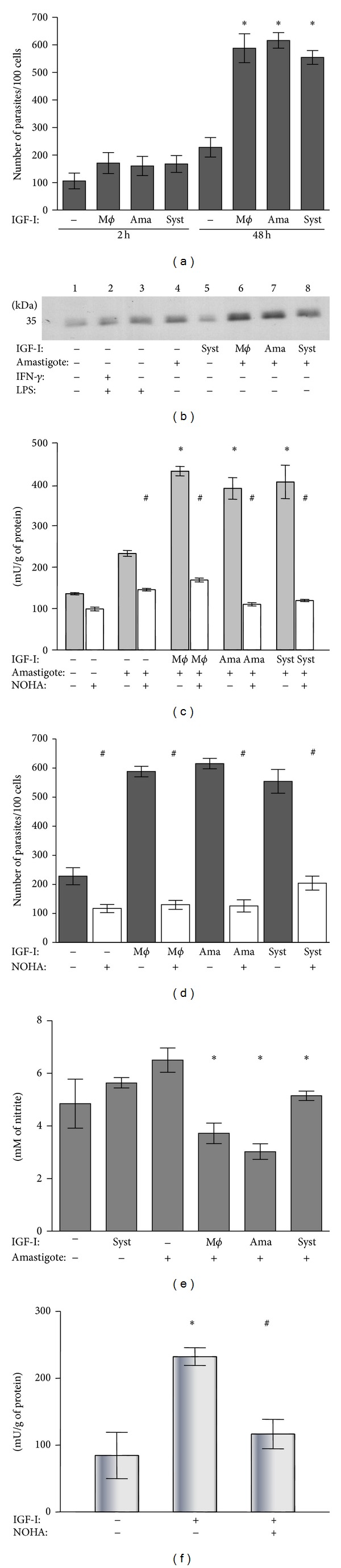
Effect of IGF-I on the* Leishmania* amastigote-macrophage interaction.** (**a) Parasite burden evaluated after 2 and 48 h of culture. (b) Arginase expression analyzed in infected macrophages at 24 h with western blotting using an anti-arginase-I antibody. (c) Arginase activity evaluated in the interaction measuring urea production with and without the arginase inhibitor NOHA. (d) Effect of inhibition by NOHA on the parasite burden in* Leishmania* amastigote-infected macrophages. (e) Measurement of nitrite production in the culture supernatant by the Griess method. (f) Effect of IGF-I on the arginase activity of* Leishmania* amastigotes. BALB/c peritoneal macrophages were infected with amastigotes of* Leishmania (L.) amazonensis.* Amastigotes (ama) or macrophages (m*ϕ*) were prestimulated for 5 min with IGF-I (50 ng/mL) before interaction or the factors were maintained in the culture system (syst) throughout the experiment period. For further details see Material and Methods Section. *P < 0.05 compared with amastigote-infected macrophage without IGF-I stimulation (ANOVA and Student Newman-Keuls tests). ^#^ *P* < 0.05 compared with culture without NOHA (ANOVA and Student Newman-Keuls tests).

Using tissue-derived* Leishmania (L.) amazonensis* amastigotes, we initially analyzed the parasitism and arginase activity in infected macrophages and extracellular amastigotes. The effects of IGF-I were assayed in the following three different ways: preincubation of amastigotes or macrophages for 5 minutes with IGF-I that was washed out or with the factor maintained in the culture medium throughout the experimental period. We observed an increase in the number of parasites within macrophages after 48 h of amastigote infection in all IGF-I stimulation conditions tested (Figure 1(a)), as observed previously with promastigotes [25]. We did not observe any effect when IGF-II was used with* L. (L) amazonensis* amastigotes (data not shown).

We observed increased arginase expression after IGF-I stimulation in infected macrophages (Figure 1(b)). To demonstrate that the enzyme activity was present after IGF-I stimulation, we measured the arginase activity by urea determination, and the activity increased after IGF-I treatment (Figure 1(c)). To confirm that the urea was derived from arginase activity, a specific arginase inhibitor (NOHA) was used to inhibit urea formation (Figure 1(c)). Urea formation increased relative to unstimulated parasites and was inhibited by NOHA. Parasitism increased with arginase activity and was inhibited by NOHA (Figure 1(d)). However, nitric oxide production decreased significantly compared to that of controls that lack IGF-I (Figure 1(e)). Neither IGF-I nor IGF-II altered the production of hydrogen peroxide in* L. (L.) amazonensis*-infected macrophages (data not shown). Furthermore, arginase activity in IGF-I-stimulated cell-free-amastigotes was also evaluated and arginase activity increased in these conditions (Figure 1(f)).

**Figure 2:**
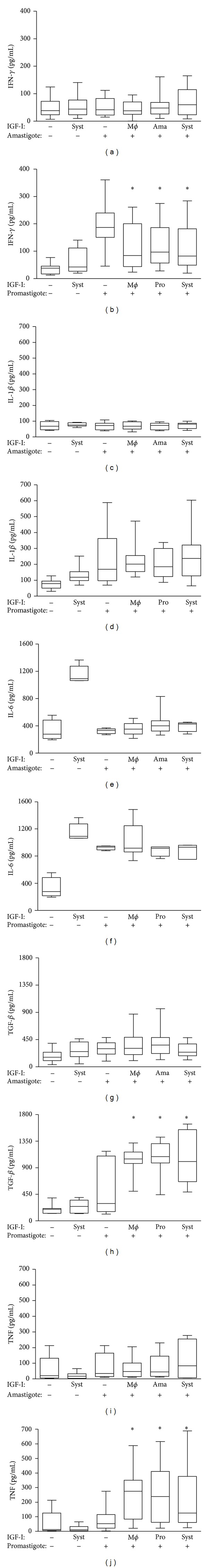
Effect of IGF-I on cytokine production by BALB/c peritoneal macrophages infected with amastigotes or promastigotes of* L. (L.) amazonensis.* Cytokine levels were determined in the culture supernatant by ELISA: IFN-*γ*: (a) and (b); IL1-*β*: (c) and (d); IL-6: (e) and (f); TGF-*β*: (g) and (h); TNF: (i) and (j). Boxes represent the median values and the 25th and 75th percentiles (3 experiments, *n* = 6). M*ϕ* = macrophage, ama = amastigote, pro = promastigote and Syst = System. *P < 0.05 compared with promastigote-infected macrophage without IGF-I stimulation. (Kruskal-Wallis test with the Student Newman-Keuls contrast post-test).

The balance between Th1 and Th2 cytokines is known to be an important determinant in the activation of the L-arginine metabolic pathways that leads to the production of nitric oxide or polyamines [26] and macrophage stimulation with IL-4, IL-10, and TGF-*β* induces arginase-I activation and leads to the increased growth of amastigotes within macrophages [30, 38]. In addition to those cytokines, the production of other inflammatory cytokines (IFN-*γ*, IL-1*β*, IL-6, IL-10, IL-l2, TGF-*β*, TNF, and PGE_2_) was analyzed in the* Leishmania amazonensis *amastigote-infected macrophages after IGF-I stimulation. IGF-I did not alter cytokine production by amastigote-infected macrophages compared to the controls without IGF-I (Figures 2(a), 2(c), 2(e), 2(g), and 2(i)). Then, as a comparison, we extended the study to analyze cytokine production by* Leishmania *promastigote-infected macrophages upon IGF-I stimulation. No alteration in IL-10, IL-12, and PGE_2_ was observed in either* Leishmania* form (data not shown); however, we observed decreased IFN-*γ* (Figure 2(b)) but increased TGF-*β* (Figure 2(h)) and TNF (Figure 2(j)) levels compared with those of the nonstimulated promastigote infected-macrophage controls. IGF-I increased production of IL-6 in uninfected macrophages, but while amastigote infection (Figure 2(e)) led to its decrease in IGF-I-stimulated cells, promastigote infection did not promote IL-6 decrease in IGF-I-stimulated cells (Figure 2(f)). IL-1*β* levels remained low in the amastigote-infected cells (Figure 2(a)) compared with the high levels in the promastigote-infected cells with or without IGF-I stimulation (Figure 2(b)).

**Figure 3:**
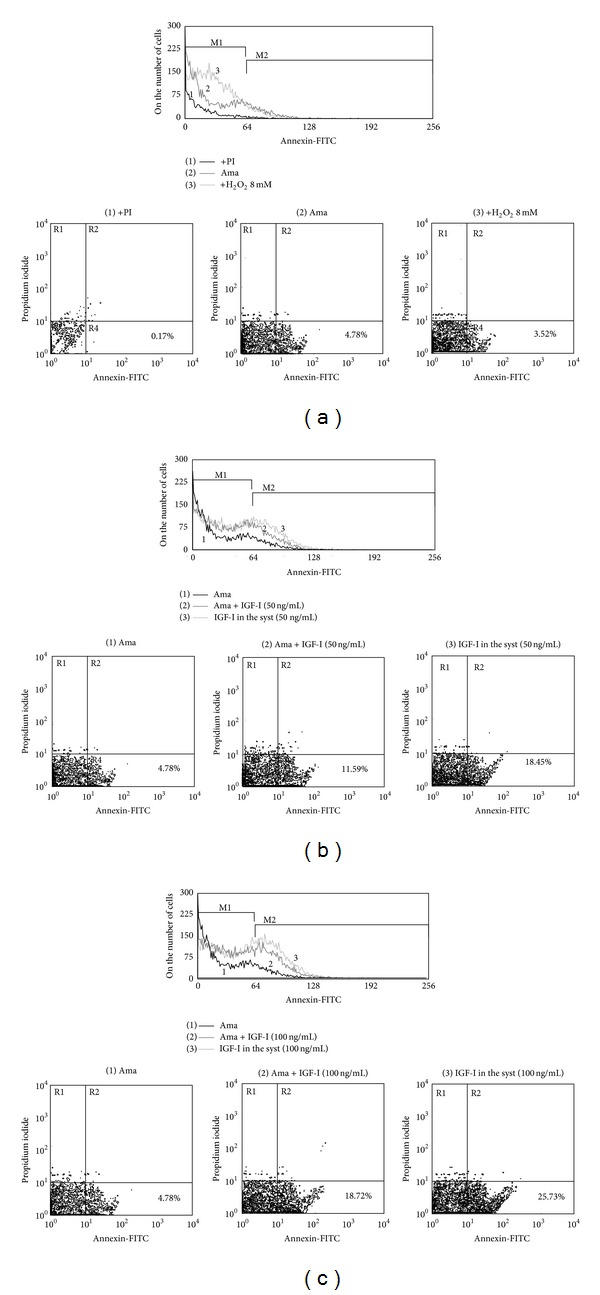
Effect of IGF-I on Annexin V binding to* L. (L.) amazonensis* after IGF-I stimulation. Annexin V-FITC binding to parasites by flow cytometry. (a) Controls. (1) Negative control-amastigotes without Annexin V-FITC label; only with propidium iodide (PI) stain; (2) Unstimulated amastigotes. (3) Positive control-amastigotes after 8 mM H_2_O_2_ stimulation. (b) IGF-I (50 ng/mL) stimulation and (c) IGF-I (100 ng/mL) stimulation. (1) Unstimulated amastigotes. (2) Amastigotes prestimulated for 5 min and maintained in culture without IGF-I for 24 h. (3) Amastigotes maintained in culture (syst) with IGF-I for 24 h. The results are representative of 3 independent experiments. Data were collected in a BD FACScalibur and analyzed by CellQuest Pro (BD Biosciences). A total of 10,000 events were harvested from each sample.

Because we did not observe effects due to IGF-I on cytokine production in the amastigote-infected macrophages, we looked for an alternative mechanism that could explain the alternative macrophages activation, focusing on PS exposure on amastigotes described before [27]. We investigated the possible effect of IGF-I on PS exposure on* L. (L.) amazonensis* after IGF-I stimulation. We used hydrogen peroxide stimulation as a positive control [35] that induced PS exposure on control promastigotes but not on control amastigotes. However IGF-I clearly increased PS exposure on amastigotes (Figure 3) but not on promastigotes (data not shown).

**Figure 4:**
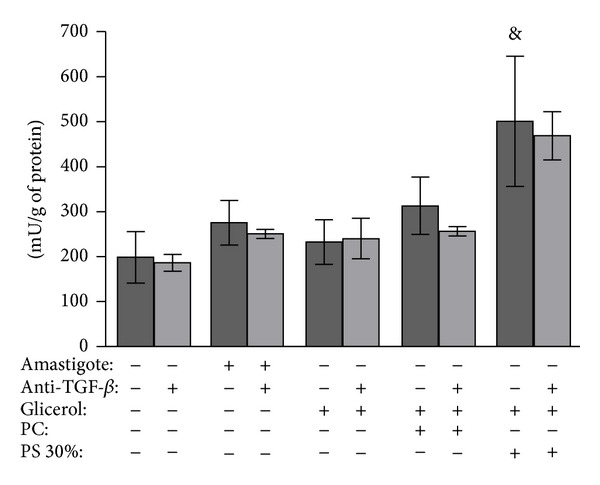
Effect of negatively charged lipid phosphatidylserine (PS)-liposomes on arginase activity of BALB/c macrophages. Peritoneal macrophages were infected with phosphatidylserine- (PS-) liposomes, phosphatidylcholine- (PC-) liposome or glycerol. Amastigotes of* Leishmania (L.) amazonensis *were used as controls. In parallel, an anti-TGF-*β* antibody was used in all interaction conditions. Macrophages were lysed and the arginase activity was determined by measuring the urea level. Assays were run in triplicate. The data are presented as the mean ± standard deviation of enzyme activity units (amount of enzyme that catalyzes the formation of 1 *μ*mol urea/min). The results are representative of two similar experiments. ^&^ *P* < 0.05 compared with control without liposome (ANOVA and Student Newman-Keuls tests).

We then determined whether PS is involved in inducing arginase activity in macrophages. To restrict the analysis to the effect of this molecule, we used a negatively charged PS-liposome instead of amastigotes. As controls, we used the neutral lipids phosphatidylcholine- (PC-) liposome and glycerol. PS-liposomes have been previously characterized and have exhibited a zeta potential of −88 mV (±20), which is a considerable negative charge when compared to PC-liposomes, which demonstrated a zeta potential of +1.25 mV (±0.2). Exposure to the PS-liposome led to an increase in macrophage arginase activity. Neither the PC-liposome nor glycerol induced arginase activity in macrophages (Figure 4). Additionally, we observed no change in the arginase activity induced by the PS-liposomes in the macrophages using an anti-TGF-*β* antibody (Figure 4).

## 4. Discussion

In the present study, we used tissue-derived amastigotes to simulate conditions similar to those in infected tissues in vivo. Initially, we observed the alternative activation of macrophages with increased arginase expression and activity and decreased nitric oxide production in* L. (L.) amazonensis *amastigote-infected macrophages, which lead to increased parasitism after IGF-I stimulation. These findings were similar to previous observations of promastigote-infected macrophages [25]. Reactive oxygen and reactive nitrogen intermediates are important nonspecific parasiticidal elements [39] and both superoxide and nitric oxide have been previously shown to be necessary for* L. (L.) amazonensis* amastigote killing within macrophages [40]. In our experiments, we evaluated hydrogen peroxide production and found that it was not increased during amastigote or promastigote infection and was not altered upon IGF-I stimulation (data not shown). However, in the present study, only the decrease in NO production upon IGF-I exposure was sufficient to increase parasitism in the macrophage. Regarding this result, we should consider the additional effect of IGF-I on activation of* Leishmania* arginase in our system. The parasite arginase is vital for its growth and arginase gene deletion has been shown to impair the progression of infection in vitro and in vivo [41, 42]. Factors that activate* Leishmania* arginase were unknown, but in our previous study with promastigotes [25] as well as in our present study with amastigotes, we show that a host IGF-I does activate parasite arginase.

Because the observed alternative macrophage activation could have been due to the modulation of cytokine production [26], we analyzed cytokine production after IGF-I stimulus [25]. In* L. (L.) amazonensis* amastigote-infected macrophages, we observed no alteration of cytokines production after IGF-I stimulation compared to unstimulated infected controls, suggesting that macrophage arginase activation due to amastigote infection does not involve the modulation of cytokine production. Interestingly, in a parallel analysis, the production of the same cytokines in* L. (L.) amazonensis* promastigote-infected macrophages showed alterations upon IGF-I stimulus. We observed decreased IFN-*γ* but increased TGF-*β* and TNF levels compared with those in unstimulated promastigote-infected macrophages, which suggest that increased TGF-*β* and decreased IFN-*γ* lead to the alternative activation of macrophages upon IGF-I stimulus in* L. (L.) amazonensis* promastigote-infected macrophages. TGF-*β* production was already increased during infection with* L. (L.) amazonensis* promastigotes as previously observed [43] and was further increased by IGF-I exposure in the present study. The presence of IFN-*γ* in the culture supernatant could have originated from contaminant lymphocytes because peritoneal macrophages were used; however, such lymphocyte contamination, if any, would be very low and the production of IFN-*γ* by macrophages has been shown previously [44]; therefore, we believe that the IFN-*γ* originated from macrophages and that its production decreased after IGF-I stimulation.

Although cytokine production was not altered by IGF-I stimulation in amastigote-infected macrophages, when compared with the promastigote-infected macrophages, we observed differences in the production of IL-1*β* and IL-6. IL-1*β* production was not altered in amastigote-infected macrophages but increased in promastigote-infected macrophages, and no changes were observed after IGF-I stimulation. We also observed increased IL-6 upon IGF-I stimulus in uninfected macrophages that remained increased at the same level in promastigote-infected cells but decreased in amastigote-infected cells to the level of the uninfected, unstimulated cells. These data suggest that amastigotes suppress cytokine production in general. In line with our results, IL-1*β* production was previously shown to be suppressed in* Leishmania donovani* amastigote-infected human monocytes [45]. Moreover,* L. major* amastigotes did not induce the production of the proinflammatory cytokine TNF and the chemokines CCL3 and CCL4, which is in contrast to the higher production during promastigote infection [22]. Similarly, with* Leishmania donovani* amastigotes, a significant proportion of genes were shown to be suppressed in host cells [46].

No alteration in cytokine production that we observed in the amastigote-infected macrophages was also observed in a study with* Leishmania major* where parasite arginase was further shown to affect host cell arginase activation [42]. This finding would be interesting to confirm with* L. (L.) amazonensis *but here we investigated PS exposure on tissue-derived* L (L.) amazonensis* amastigotes as it relates to the progression of the infection and is called apoptotic mimicry [27]. The induction factor was previously unknown, and because IGF-I is present in tissue fluids [47] and within macrophages [48] which is the amastigotes niche, we considered the possible effect of this factor on PS exposure in amastigotes. In fact, IGF-I stimulation induced PS exposure on* L. amazonensis* amastigotes but, interestingly, not on promastigotes. A recent publication regarding* Leishmania* promastigotes shows the absence of PS in this form of the parasite [49] although in our experiment with promastigotes the hydrogen peroxide induced exposure of PS or some similar molecule that has bound to anexin V (data not shown). However, we should emphasize that apoptotic mimicry [27] and the present results refer to PS exposure on amastigotes, and the presence of PS is considered to be likely in other growth phases by the same authors [49].

In the study on apoptotic mimicry, increased parasite growth was attributed to TGB-*β* production [27]; however, in the present study, we explored the effect of PS exposure on arginase activity in macrophages. Using PS-liposomes to restrict the analysis to this particular molecule, we were able to show that in fact PS-liposomes but not the control PC-liposomes led to increased arginase activity in the macrophages. Furthermore, neutralization of TGF-*β* using an anti-TGF-*β* antibody had no effect on arginase activity. Of note, PS-liposomes were previously shown to have an effect on L-arginine metabolism, leading to a decrease in NO production by lipopolysaccharide-stimulated macrophages [50]. In the present study, we also observed decreased nitric oxide production but increased arginase activity after IGF-I stimulation in amastigote-infected cells, suggesting the preferential activation of the alternative macrophage pathway, possibly due to PS exposure on amastigotes after stimulation. Further studies must be performed to clarify how PS-liposomes activate macrophage arginase, but because arginase is a trimeric metalloenzyme that contains a binuclear manganese cluster in the active site [51], we can speculate that the negative charge of the PS-liposome could carry manganese through cell membrane when PS-liposomes are internalized by macrophages [37], providing manganese to the enzyme and activating it.

Studies using mouse [52] and human cells [53] show an increased* Leishmania* parasite burden when apoptotic neutrophils are ingested by macrophages. This increased burden occurs when using neutrophils derived from the* Leishmania major*-susceptible BALB/c mouse strain. Considering the data from the present study, we can speculate that this phenomenon may be related to the arginase activation induced by PS exposure on apoptotic cells. In the above-mentioned studies, the effect was related to the induction of PGE_2_ and TGF-*β* production [52, 53]; however, our data contradict this observation as a possible mechanism in the present experiments.

The present data suggest that in* L. (L.) amazonensis* amastigote-infected macrophages, IGF-I induces arginase activity directly on amastigotes and also in macrophages. The cytokine production data suggest that amastigote infection leads to the suppression of cytokine production with no contribution to macrophage activation upon IGF-I stimulation. A phenomenon that is seemingly peculiar to* L. (L.) amazonensis* is the induction of PS exposure on amastigotes, which was previously observed on tissue-derived amastigotes and was induced by IGF-I and contributes to the progression of the infection in the present study. Finally, we should emphasize that the activation of macrophage arginase by PS-liposomes leads to the alternative activation of macrophages through cytokine-independent mechanisms.
